# Homology Models of Melatonin Receptors: Challenges and Recent Advances

**DOI:** 10.3390/ijms14048093

**Published:** 2013-04-12

**Authors:** Daniele Pala, Alessio Lodola, Annalida Bedini, Gilberto Spadoni, Silvia Rivara

**Affiliations:** 1 Dipartimento di Farmacia, Università degli Studi di Parma, Parco Area delle Scienze 27/A, 43124 Parma, Italy; E-Mails: daniele.pala@nemo.unipr.it (D.P.); alessio.lodola@unipr.it (A.L.); 2 Dipartimento di Scienze Biomolecolari, Università degli Studi di Urbino “Carlo Bo”, Piazza Rinascimento 6, I-61029 Urbino, Italy; E-Mails: annalida.bedini@uniurb.it (A.B.); gilberto.spadoni@uniurb.it (G.S.)

**Keywords:** melatonin receptors, MT_1_, MT_2_, homology modeling, structure-activity relationships, docking, molecular dynamics simulations

## Abstract

Melatonin exerts many of its actions through the activation of two G protein-coupled receptors (GPCRs), named MT_1_ and MT_2_. So far, a number of different MT_1_ and MT_2_ receptor homology models, built either from the prototypic structure of rhodopsin or from recently solved X-ray structures of druggable GPCRs, have been proposed. These receptor models differ in the binding modes hypothesized for melatonin and melatonergic ligands, with distinct patterns of ligand-receptor interactions and putative bioactive conformations of ligands. The receptor models will be described, and they will be discussed in light of the available information from mutagenesis experiments and ligand-based pharmacophore models. The ability of these ligand-receptor complexes to rationalize structure-activity relationships of known series of melatonergic compounds will be commented upon.

## 1. Introduction

**Figure 1:**
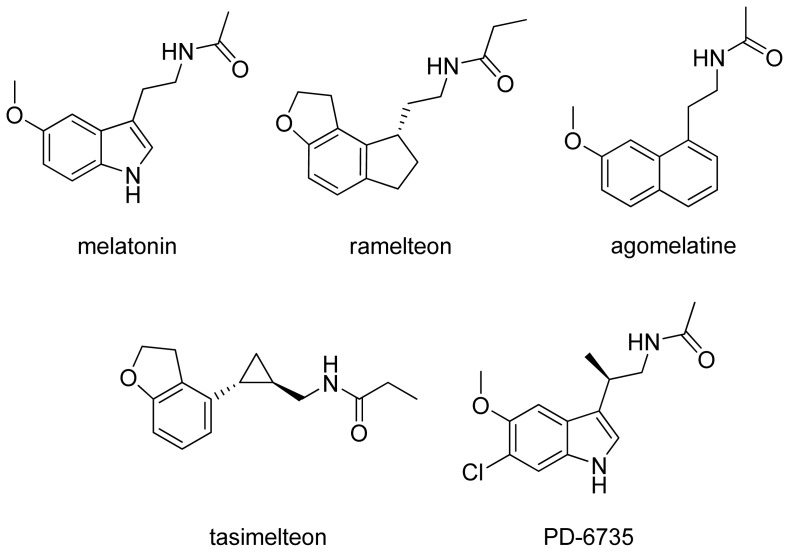
Melatonin and melatonergic agonists approved or currently tested in clinical trials.

Melatonin (*N*-acetyl-5-methoxytryptamine, Figure 1) is a tryptophan-derived neurohormone secreted by the pineal gland following a circadian rhythm and with peak concentrations at night. After its isolation and chemical characterization in 1959 [1], several studies were reported that aimed at clarifying the physiological and behavioral responses exerted by this endogenous substance. Nowadays, after more than 50 years of research, a growing body of evidence supports the key role of melatonin in the transduction of photoperiodic information, such as the entrainment and the regulation of circadian rhythms [2,3]. However, besides its well-known chronobiological and sleep-promoting effects, melatonin was found to be involved in a variety of other pathophysiological processes, including the modulation of immune and cardiovascular responses, bone formation, tumor suppression and type 2 diabetes. Furthermore, a number of experimental evidences highlighted the role of melatonin in antioxidant and neuroprotective processes [4–6]. Due to its pleiotropic activity, administration of melatonin has been proposed as a therapeutic strategy for the treatment of a variety of pathological conditions, including sleep disturbances, depression, cancer, stroke and epilepsy [7]. However, its poor pharmacokinetic profile, e.g., low solubility and short half-life, prompted the design of synthetic ligands endowed with improved pharmacokinetic and pharmacodynamic properties. Ramelteon and agomelatine have been recently approved for the treatment of insomnia and of major depression, respectively and tasimelteon and PD-6735 are currently being evaluated for the treatment of circadian rhythm sleep disorders (Figure 1) [8].

In mammals, melatonin exerts many of its actions through the activation of two G protein-coupled receptors (GPCRs), named MT_1_ and MT_2_[9]. Melatonin also binds with lower affinity to the so-called MT_3_ binding site, which has been characterized as an enzyme belonging to the quinone reductase family [10]. In humans, MT_1_ and MT_2_ receptors are expressed in several areas of the central nervous system, such as the suprachiasmatic nucleus, substantia nigra and nucleus accumbens, as well as in different peripheral tissues, including immune cells, retina and coronary arteries [4]. Since the cloning of MT_1_ and MT_2_ subtypes in the 1990s [11,12], a number of studies were reported aimed at characterizing their different behavioral responses. Thus far, experimental evidence indicates that activation of MT_1_ receptors reduces neuronal firing within the suprachiasmatic nucleus, induces vasoconstriction and inhibits hormone secretion, whereas activation of the MT_2_ subtype produces a phase shift in circadian rhythms, promotes non-rapid eye movement (NREM) sleep, induces splenocyte proliferation and vasodilation and regulates oxytocin receptor gene expression [9,13]. However, despite these efforts, the pathophysiological role of both receptors has yet to be fully elucidated. To this end and to develop novel therapeutic agents, research activity has been focused on the discovery of new compounds able to interact with these receptors. Nowadays, a number of melatonergic ligands are available, characterized by high binding affinity, with different selectivity for the two receptor subtypes and intrinsic activity [14,15]. The design of melatonergic ligands was mainly driven by ligand-based techniques, such as pharmacophore models and quantitative structure-activity relationship (QSAR) studies. Conversely, application of structure-based design was hampered by the lack of experimentally determined structures of MT_1_ and MT_2_ receptors. In this scenario, homology modeling was widely applied to investigate the key determinants for ligand binding at a molecular level.

Prediction of the three-dimensional (3D) structure of melatonin receptors represents a complex issue due to different elements, including (i) the lack of a suitable template structure, (ii) the lack of a validated pattern of ligand-receptor interactions and (iii) the nature of the endogenous ligand and the amino acid composition of the putative binding site. The first issue is related to the disparity between the number of identified GPCR sequences and the number of experimentally determined structures available to date. In fact, the GPCR X-ray structures currently deposited in the Protein Data Bank cover only ~2% of nearly 800 GPCR sequences thus far identified in the human genome. As a consequence, closely related templates are available only for a restricted subset of receptors, while the majority of GPCRs still has to be modeled using “structurally remote” templates. Recent studies on GPCR homology models clearly highlighted that the reliability of a receptor model strongly depends on its degree of sequence identity with the template receptor. Accordingly, while the availability of an experimentally determined GPCR structure having a sequence identity >30%–35% to the target usually provides reliable models, “remote-template” homology modeling procedures (*i.e.*, when the identity is below 30%) often fail to produce accurate receptor models [16–19]. MT_1_ and MT_2_ receptors share a sequence identity lower than 30% with crystallized GPCRs. In particular, sequence identity percentages calculated within transmembrane (TM) domains range from 19% to 29%, which decrease to 8%–28% in the binding site region. Another issue making melatonin receptor modeling even more challenging is the lack of well-characterized ligand–receptor interactions. Indeed, although site-directed mutagenesis studies were conducted on a number of different residues, spanning all TM domains of MT_1_ and MT_2_ receptors, an unambiguous binding mode for melatonin has not been identified. The difficulties in MT_1_ and MT_2_ receptor modeling could be also related to the physicochemical properties of the endogenous ligand, as well as to the amino acid composition of the putative binding pocket. Both melatonin receptors lack the pattern of polar and charged binding site residues that anchor polar neurotransmitters [20]. Due to the presence of such a hydrophobic environment and to the remarkable lipophilic character of melatonin, it is unlikely that melatonergic compounds can form strong polar contacts within the binding region.

Although the modeling of melatonin receptors cannot rely on close structural templates and on a validated pattern of ligand-receptor interactions, a number of MT_1_ and MT_2_ 3D models have been reported and the binding mode of different ligands has been proposed. Most of these receptor models were built before 2007, when the first X-ray structure of a druggable GPCR, the β_2_ adrenergic receptor, was released. Consequently, the majority of these models were built starting from the structure of bovine rhodopsin, which was the only structural template available. Only recently, homology models were built based on the newly released X-ray structures of aminergic receptors or of GPCR structures crystallized in their fully-activated state. Melatonin receptor models were usually applied to propose a binding scheme for melatonin and to rationalize structure-activity relationships (SARs) or mutagenesis data. The recent advances of computational techniques, as well as the increase of available ligand-based information also allowed the building of SAR- and mutagenesis-consistent receptor models, which were applied either to describe agonist binding at a molecular level or to assess the molecular basis of ligand selectivity or intrinsic activity.

This review provides an overview on published MT_1_ and MT_2_ receptor homology models. The description of the 3D models and of their ligand binding scheme is preceded by two paragraphs summarizing the main structural elements of the MT_1_ and MT_2_ receptors and the available mutagenesis data.

## 2. MT_1_ and MT_2_ Melatonin Receptors

### 2.1. Structure

Within the GPCR superfamily, melatonin receptors are members of the so-called “class A” family, which was initially defined by Kolakowski [21] and subsequently revised by the NC-IUPHAR [22]. The class A of GPCRs was also re-named as the “rhodopsin family” according the GRAFS classification scheme [23]. MT_1_ and MT_2_ receptors are characterized by the peculiar sequence motif characteristic of the rhodopsin family, constituted by seven TM-spanning helices connected by three intracellular (ICL1, ICL2, ICL3) and three extracellular (ECL1, ECL2, ECL3) loop segments. Both receptor subtypes show a pattern of highly conserved residues and motifs shared by all GPCRs belonging to this class, which can be found in TM sequences and in loop regions. These conserved sequence “fingerprints” of rhodopsin-like receptors have been extensively exploited during homology modeling studies to guide the alignment between template and melatonin receptor sequences.

**Figure 2:**
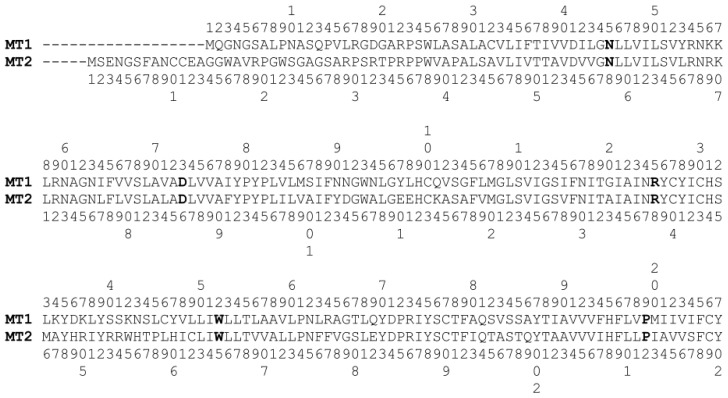

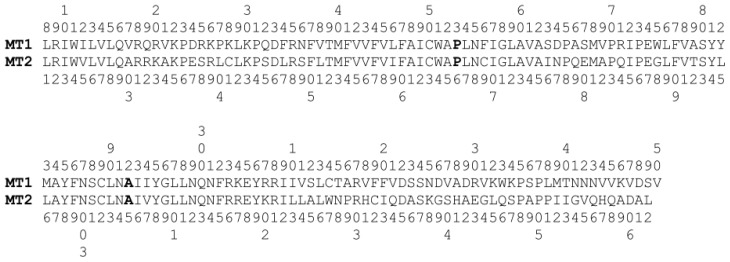
Amino acid sequences of human MT_1_ and MT_2_ receptors. Amino acids at positions X.50 of each transmembrane helix are indicated in bold.

**Figure 3:**
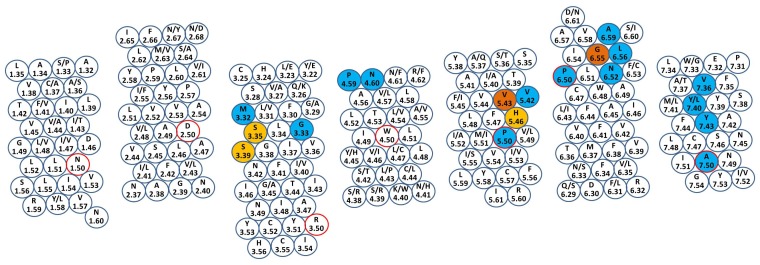
Snake plot of the putative transmembrane helices of human MT_1_ and MT_2_ receptors. For each amino acid, one letter code (if two codes are present, the first refers to MT_1_, the second to MT_2_) and Ballesteros-Weinstein numbering are reported. Amino acids X.50 are circled in red. Filled circles refer to amino acids for which mutagenesis data are discussed in the text; orange: MT_1_ mutant; blue: MT_2_ mutant; yellow: MT_1_ and MT_2_ mutants. The picture is a modification of a snake plot made with Snake-Plot-Designer (www.ssfa-7tmr.de/ssfe).

MT_1_ and MT_2_ receptors show a pattern of highly conserved positions within TM domains, *i.e.*, Asn45/58^1.50^, Asp73/86^2.50^, Arg125/138^3.50^, Trp152/165^4.50^, Pro199/212^5.50^, Pro253/266^6.50^ and Ala292/305^7.50^ (residue numbers of MT_1_ and MT_2_ receptors are separated by a slash and superscripts refer to Ballesteros-Weinstein numbering [24]) (Figure 2). Besides the X.50 positions, other highly conserved residues have been identified among class A GPCRs, which could be exploited in modeling studies of melatonin receptors to assist the template-receptor alignment [25]. For example, the TM1 domain of both melatonin receptors shows the presence of a glycine (Gly44/57^1.49^) and a leucine (Leu47/60^1.52^), which have also been found in 67% and 61% of rhodopsin-like GPCRs, respectively [26] (Figure 3). Furthermore, a conserved hydrophobic LAXADL motif located within TM2 of class A GPCRs could be also recognized within TM2 of MT_1_ and MT_2_ subtypes, spanning from Leu69/82^2.46^ to Leu74/87^2.51^. A cysteine residue could be identified at the *N*-terminus of TM3 in both melatonin receptors (Cys100/113^3.25^), which is conserved in more than 85% of rhodopsin-like GPCRs. Interestingly, crystallographic structures of GPCRs show that Cys^3.25^ is involved in a disulfide bond with another conserved cysteine located in ECL2, providing an overall stabilization of the receptor extracellular region. The *C*-terminus of TM3 of class A GPCRs is characterized by the conserved D(E)RY motif. The arginine at position 3.50 is involved in the formation of the so-called “ionic-lock”, *i.e.*, a salt bridge interaction between the positively charged Arg^3.50^ and a negatively charged amino acid located at position 6.30 of TM6, which contributes to the stabilization of the inactive form of the receptor. In MT_1_ and MT_2_ receptors, the conserved acidic residue located at position 3.49 is replaced by an asparagine (Asn124/137^3.49^), leading to a NRY sequence. In TM4, the proline residue located at the *C*-terminus (Pro161/174^4.59^) is shared by more than 60% of rhodopsin-like receptors. The TM5 segment of class A GPCR is characterized by two conserved aromatic amino acids, namely Phe^5.47^ and Tyr^5.58^, which can be found in 61% and 75% of class A GPCRs, including MT_1_ and MT_2_ subtypes (Phe196/209^5.47^ and Tyr207/220^5.58^). Together with TM3, TM6 and TM7 domains of class A GPCRs are also known to regulate the receptor functional state. Site-directed mutagenesis studies, as well as the newly released structures of GPCRs crystallized in their fully activated conformation, clearly highlight the importance of the CWXP stretch on TM6 and of the NPXXY sequence on TM7 in triggering the specific conformational changes associated with receptor activation. Interestingly, while the former motif is strictly maintained in melatonin receptors, which show a CWAP sequence spanning from Cys250/263^6.47^ to Pro253/266^6.50^, the highly conserved proline residue of the latter NPXXY pattern is replaced by an alanine in both subtypes (Ala292/305^7.50^), leading to a NAXXY motif.

Besides TM domains, conserved residues and motifs can be identified also within the extracellular and intracellular regions of rhodopsin-like GPCRs. For example, a WXFG domain has been found in the ECL1 segment of 80% of class A GPCRs [27], which is probably involved in the transmission of the ligand binding signal to the TM portions. In available crystallographic structures, the tryptophan residue forms hydrophobic interactions with the cysteine residues of the disulfide bridge connecting the tip of TM3 to the ECL2 segment. MT_1_ and MT_2_ receptors show a WXLG motif within the ECL1 region (Trp93/106-Gly96/109), which retains the tryptophan and the glycine residues identified in the highly conserved WXFG sequence. Another conserved amino acid outside the TM domains is a leucine residue located in the ICL1 segment [28,29]. In a number of different crystal structures, this leucine forms a network of interactions with neighboring residues located at the intracellular ends of TM1 and TM2, as well with a set of amino acids belonging to the last α-helix segment that runs parallel to the membrane plane (the so-called “helix 8”). This leucine is also conserved in MT_1_ and MT_2_ subtypes (Leu58/71), where it probably favors the helix packing in the same manner as observed for other rhodopsin-like receptor structures.

### 2.2. Mutagenesis Data

While sequence-based information provided by rhodopsin-like GPCRs can be exploited to guide the modeling of melatonin receptor 3D structures, the identification of the melatonin binding site is still an open issue. Indeed, although a number of mutagenesis studies have been carried out for both MT_1_ and MT_2_ subtypes, information provided by these investigations does not allow the identification of a definite subset of residues involved in specific ligand-receptor interactions. Thus far, site-directed mutagenesis studies on MT_1_ and MT_2_ subtypes were performed on a number of residues located on TM3, TM4, TM5, TM6 and TM7. The putative binding cavity outlined by these studies can be roughly superposed to the prototypic binding site observed in a variety of GPCR crystal structures, which spans from TM3 to TM7 and lays in the extracellular portion of the helical bundle. His195/208^5.46^ was initially evaluated as an anchor point for melatonin. Interestingly, all the members of the melatonin receptor subfamily (*i.e.*, melatonin type 1 and melatonin-like (GPR50) receptors from different species) exhibit a histidine residue at position 5.46, and a number of experimental studies reported a key role for this amino acid [30–32]. Indeed, substitution of His^5.46^ led to a decrease of binding affinity for melatonin and other ligands at both receptor subtypes. In GPCR X-ray structures, residue 5.46 is located one turn above the superconserved Pro^5.50^ and often points towards the stretches of TM3 and TM4 facing the binding site (e.g., His211^5.46^ of rhodopsin and Asn198^5.46^ of the H_1_ histamine receptor). Site-directed mutagenesis studies performed on a number of GPCRs further confirmed a crucial role of position 5.46 in ligand stabilization, as well as in receptor activation [33–35]. Mutagenesis studies also highlighted that four other residues are fundamental for melatonin binding to the MT_1_ subtype, namely Ser110^3.35^, Ser114^3.39^, Val192^5.43^ and Gly258^6.55^. The two serines located on TM3 are fully conserved within the melatonin receptor subfamily and their mutation to alanines brought from a four- to a nine-fold decrease in melatonin binding affinity [36]. Serine 3.39 is located in the middle of TM3 in many GPCR structures, close to the Asp^2.50^ side chain, and it is conserved in more than 70% of rhodopsin-like receptors. A recent crystallographic study revealed that Ser^3.39^ in the A^2A^ adenosine receptor (Ser91^3.39^) is responsible for the stabilization of the sodium ion [37], which acts as an allosteric modulator for this receptor subtype [38]. On the other hand, Ser^3.35^ can be detected in only 21% of class A GPCRs, and it is located one turn above position 3.39, at the same level of the tryptophan residue (Trp^6.48^) belonging to the CWXP domain. Intriguingly, residue 3.35 was found to play a crucial role in a number of different GPCRs, since mutation of this amino acid in the β_2_ adrenergic receptor (Cys116^3.35^) and in the nociceptin receptor (Asn133^3.35^) brought to constitutively active proteins [39,40]. Similarly to His^5.46^, Ser^3.35^ and Ser^3.39^, also Val^5.43^ (Val192^5.43^ in the MT_1_ receptor) is fully conserved among the melatonin receptor subfamily. Mutagenesis studies performed on the ovine MT_1_ receptor clearly showed that substitution of Val208^5.43^ to either alanine or leucine led to a decrease in the binding affinity of melatonin and of other melatonergic ligands [30]. X-ray structures of class A GPCRs show that this residue is involved in the definition of the binding cavity and forms an extensive network of interactions with surrounding residues (e.g., Ser212^5.43^ of the β_1_ adrenergic receptor and Phe182^5.43^ of the A_2A_ adenosine receptor). The importance of residue 5.43 for ligand binding and receptor stabilization is also sustained by site-directed mutagenesis experiments performed on a number of rhodopsin-like receptors [41,42]. Mutation of Gly258^6.55^ to threonine brought to a severe loss of 2-iodomelatonin binding affinity and to a remarkable impairment of signal transduction [43,44]. In most GPCR structures, residue 6.55 is located at the *C*-terminus of TM6, near the extracellular edge of the helical bundle. Visual inspection of available X-ray structures revealed that residues located at position 6.55 form an extensive network of interactions with co-crystallized ligands, as well as with neighboring residues (e.g., Asn253^6.55^ in the A_2A_ adenosine receptor, Asn293^6.55^ in the β_2_ adrenergic receptor and Ile294^6.55^ in the κ-opioid receptor). In addition, mutagenesis experiments performed on different rhodopsin-like GPCRs confirmed a crucial role of position 6.55 in ligand binding and in receptor activation [45–47].

Compared to the MT_1_ receptor, mutagenesis experiments on the MT_2_ subtype were conducted on a wider array of residues, providing an in-depth analysis of the TM domains thought to define the putative binding site region. Indeed, a two- and three-fold decrease in melatonin binding affinity was observed due to the mutation of Met120^3.32^ and Gly121^3.33^ to alanine, respectively [48]. Substitution of the asparagine residue at position 4.60 (Asn175^4.60^), which is highly conserved within the melatonin receptor subfamily, with alanine led to a four-fold decrease in melatonin binding affinity [32]. More interestingly, mutation of serine residues located at positions 3.35 (Ser123^3.35^) and 3.39 (Ser127^3.39^) did not produce the remarkable loss of binding affinity seen in the MT_1_ receptor [32]. Although only sparse indications were given for residues laying on TM3 and TM4, mutagenesis experiments allowed the identification of a wide subset of important residues mainly located on TM5, TM6 and TM7, including Val204^5.42^, Asn268^6.52^, Leu272^6.56^, Ala275^6.59^, Val291^7.36^, Leu295^7.40^ and Tyr298^7.43^. Position 5.42 is located four residues (*i.e.*, one α-helix turn) before the conserved His^5.46^, and in GPCR X-ray structures, it often participates in direct ligand-receptor interactions within the binding site region (e.g., Ser203^5.42^ and Ser211^5.42^ of the β_2_ and β_1_ adrenergic receptors, respectively). Interestingly, position 5.42 is occupied by hydrophobic residues, valine or isoleucine, in all receptors belonging to the melatonin receptor subfamily, probably due to the lipophilic character of the natural ligand. Mutation of Val204^5.42^ to alanine brought to a complete loss of 2-iodomelatonin specific binding [49]. Asn268^6.52^ is fully conserved among all the members of the melatonin receptor subfamily, and it was found to play a crucial role in ligand binding. Indeed, only the conservative mutation from asparagine to glutamine maintained 2-iodomelatonin binding [48]. Conversely, mutation of Asn268^6.52^ to the non-polar alanine and leucine or to the negatively charged aspartic acid completely abolished agonist binding to the MT_2_ subtype [48]. Position 6.52 is occupied by either a phenylalanine or a histidine in more than 45% of class A GPCRs, whereas only 14% of them carry an asparagine. While an aromatic residue at position 6.52 often stabilizes the co-crystallized ligand through either edge-to-face interactions (e.g., Phe290^6.52^ of the β_2_ adrenergic receptor and Phe307^6.52^ of the β_1_ adrenergic receptor) or hydrophobic contacts (e.g., His250^6.52^ of the A_2A_ adenosine receptor), an asparagine residue can form extensive hydrogen bond contacts with the ligand and with surrounding residues (e.g., Asn404^6.52^ and Asn507^6.52^ of the M2 and M3 muscarinic receptor, respectively). Mutations of either Leu272^6.56^ to alanine or of Ala275^6.59^ to isoleucine were shown to be detrimental for binding affinity, leading to a complete loss of 2-iodomelatonin specific binding [48,49]. Conversely, the mutation of Ala275^6.59^ to valine did not influence 2-iodomelatonin binding [48]. In crystallographic GPCR structures, positions 6.56 and 6.59 are located at the *C*-terminus of TM6, near the extracellular region of the TM bundle. In particular, position 6.56 is occupied by hydrophobic residues, such as leucine, isoleucine, phenylalanine or valine in more than 70% of class A GPCRs and in all the receptors of the melatonin subfamily. Compared to position 6.56, residues at position 6.59 are more solvent-exposed, and they often form extensive hydrophobic interactions with amino acids laying at the *N*-terminus of TM5 (e.g., Val297^6.59^ of the β_2_ adrenergic receptor and Phe257^6.59^ of the A_2A_ adenosine receptor) and with residues located at the *C*-terminal stretch of ECL2 (e.g., Phe276^6.59^ of rhodopsin). Site-directed mutagenesis studies highlighted a key role of this residue in either ligand stabilization or G protein coupling in a number of different rhodopsin-like receptors, including the neuropeptide Y, the A_2A_ adenosine and the M1 muscarinic receptors [50–52]. Three different amino acids were identified in the extracellular half of TM7, which were shown to influence ligand binding to the MT_2_ subtype. Mutation of Val291^7.36^, Leu295^7.40^ or Tyr298^7.43^ to alanine disrupted agonist binding to the MT_2_ receptor [48,49]. A visual inspection of GPCR X-ray structures shows that residues 7.36, 7.40 and 7.43 are located in different regions of the TM7 domain, separated by approximately one helix turn. Residues 7.36 lay at the *N*-terminus of TM7 within the binding crevice, where they often form extensive interactions with the co-crystallized ligand (e.g., Tyr271^7.36^ of the A_2A_ adenosine receptor). In all the members of the melatonin receptor subfamily, this position is occupied by hydrophobic residues, including valine and leucine. Amino acids located at position 7.40 do not point towards the binding cavity, but they often occupy a narrow interface between TM1, TM2 and TM7, where they can form contacts with neighboring side chains (e.g., Trp313^7.40^ of the β_2_ adrenergic receptor, Phe293^7.40^ of rhodopsin and Trp455^7.40^ of the H_1_ histamine receptor). Amino acids at position 7.43 are often enclosed in the helical bundle, pointing towards the binding site crevice. Due to the peculiar orientation of residues 7.43, they can participate in ligand-receptor interactions (e.g., His278^7.43^ of the A_2A_ adenosine receptor and Lys296^7.43^ of rhodopsin) or in inter-helical contacts with surrounding amino acids (e.g., Tyr316^7.43^ of the β_2_ adrenergic receptor and Tyr430^7.43^ of the M2 muscarinic receptor). A tyrosine residue is present at position 7.43 in all the receptors belonging to the melatonin subfamily. Interestingly, a number of site-directed mutagenesis studies performed on different rhodopsin-like GPCRs highlighted a key role of positions 7.36, 7.40 and 7.43 in either ligand stabilization or receptor activation [47,53–57]. Besides the TM domains, a key residue involved in ligand stabilization has been found at the *N*-terminus stretch of the ECL2 segment, before the conserved cysteine residue of class A GPCRs. Substitution of Tyr188 with either alanine or phenylalanine completely abolished 2-iodomelatonin specific binding [48].

As previously outlined, although mutagenesis studies were conducted on a wide set of amino acids of MT_1_ and MT_2_ receptors, an unambiguous binding mode for melatonin is still missing. Indeed, it is not possible to distinguish between amino acids directly interacting with the ligands and those whose mutation leads to a modification of the receptor state, like, for example, a structural rearrangement only indirectly affecting ligand binding affinity. Moreover, only agonist compounds are currently available as radiolabelled ligands (*i.e.*, 2-[^125^I]-iodomelatonin and [^3^H]-melatonin). As the high-affinity binding of agonists depends on both ligand recognition and receptor activation, it is difficult to know which process is actually affected by a mutation. Experimental affinity of a labelled antagonist would, in this sense, provide more direct information on ligand recognition at its binding site. The putative binding cavity of MT_1_ and MT_2_ receptors is characterized by a number of hydrophobic residues. This makes the outcome of mutagenesis experiments even more complicated for interpretation, since the disruption of a weak hydrophobic interaction usually brings a limited loss in binding affinity. As a consequence of the number and the diverse location of residues important for binding affinity, melatonin interacts only with a small subset of these amino acids in the proposed binding modes.

## 3. MT_1_ and MT_2_ Melatonin Receptor Models

During the last two decades, a number of MT_1_ and MT_2_ homology models have been reported, which were built starting from different GPCR X-ray crystal structures. In this section, we review these receptor models along with a brief description of the overall homology modeling workflow and of the computational techniques applied to refine the model structure. The binding modes proposed for melatonin and/or for other melatonergic ligands are discussed in light of available information, such as pharmacophore models, mutagenesis data and consistency with SAR studies.

### 3.1. MT_1_ Receptor Models

**Figure 4:**
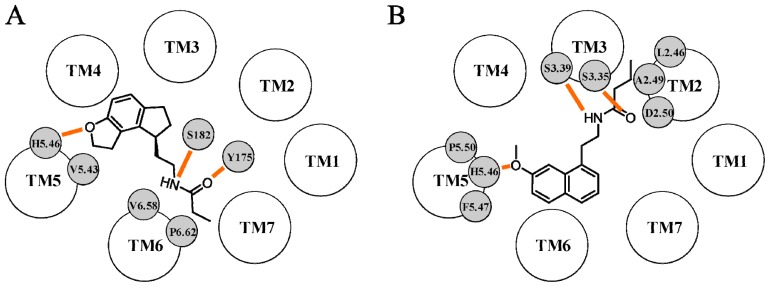
Representation of the binding scheme proposed by Uchikawa *et al.* for ramelteon (**A**) and by Ivanov *et al.* for a naphthalene agonist (**B**) within the MT_1_ binding site. Hydrogen bond interactions are depicted with orange lines.

The first MT_1_ receptor model based on the X-ray crystal structure of a GPCR was described by Uchikawa *et al.* in 2002 [58]. They used the first available crystal structure of rhodopsin (PDB ID: 1F88 [59]) to build the TM domains and the ECL2 portion of the MT_1_ receptor. Since the initial geometry of ECL2 was directly derived from the corresponding segment of the rhodopsin structure, this loop protruded into the helical bundle and sealed the putative binding cavity of the MT_1_ structure, hampering the accommodation of melatonergic ligands. Thus, a simulated annealing procedure was applied to induce a structural rearrangement of the ECL2 portion, favoring the accommodation of melatonergic derivatives. Ramelteon (Figure 1, p*K*_i_ (MT_1_) = 10.9) was docked into the refined model to identify the structural features able to confer binding affinity. In its best docking pose, it formed a network of hydrogen bonds and hydrophobic interactions with residues located on TM5, TM6 and ECL2 (Figure 4A). In particular, the oxygen atom of the dihydrofuran ring was hydrogen bonded to His195^5.46^ on TM5, whereas the adjacent carbon atom interacted with the side chain of Val192^5.43^. These two residues had been previously indicated as important for melatonin stabilization at the MT_1_ subtype [30,31]. The indane core laid at the bottom of the binding cavity and was surrounded by a number of hydrophobic residues. On the basis of this docking result, the authors hypothesized that substitution of the NH group of the indole ring of melatonin with the more lipophilic methylene unit of the indane one could explain the six-fold increase in binding affinity compared to melatonin. The amide chain was stabilized by several contacts with residues located on TM6 and ECL2. Indeed, Val261^6.58^ and Pro265^6.62^ formed hydrophobic interactions with the alkyl portion of the propionamide group and the amide oxygen and hydrogen atoms interacted with Tyr175 and Ser182, respectively, located on ECL2. Although no mutagenesis data are available for positions 6.58 and 6.62, the importance of the ECL2 portion in ligand binding to the MT_1_ receptor has been assessed in studying chimeric melatonin receptors [44]. Ramelteon did not interact with the two serine residues located on TM3, Ser110^3.35^ and Ser114^3.39^, which were shown to be fundamental for ligand stabilization.

The first complete model of the MT_1_ receptor was reported a few years later by Ivanov *et al.*[60]. In this study, the rhodopsin X-ray structure solved at 2.6 Å (PDB ID: 1L9H [61]) was initially used to build the TM portions and the first two extracellular loops of the MT_1_ receptor, together with the conserved disulfide bridge between Cys100^3.25^ and Cys177. The other loop segments were subsequently modeled on the energy-minimized receptor conformation. To investigate the molecular basis of ligand recognition, five agonists were manually docked into the putative binding cavity. Information from mutagenesis experiments was used as guidance for the initial ligand placement. Docking results obtained for melatonin, 2-iodomelatonin and the naphthalene derivative depicted in Figure 4B outlined a common binding mode, in which the methoxy group was hydrogen bonded to His195^5.46^ and the amide oxygen and hydrogen atoms interacted with Ser110^3.35^ and Ser114^3.39^, respectively. The methyl group of the methoxy substituent was stabilized by a number of hydrophobic interactions with neighboring residues, including Ile115^3.40^, Phe196^5.47^ and Pro199^5.50^. While the role of His195^5.46^, Ser110^3.35^ and Ser114^3.39^ in ligand stabilization had been assessed, no mutagenesis data are available for positions 3.40, 5.47 and 5.50. Docking results also showed that the 2-iodine atom of 2-iodomelatonin occupied a cleft surrounded by Trp251^6.48^ and Leu254^6.51^, located on TM6. Although no mutagenesis experiments were conducted on Trp251^6.48^ of the conserved CWXP motif, the corresponding tryptophan residue of the MT_2_ receptor subtype (Trp264^6.48^) was shown to be important for ligand stabilization [32]. Interestingly, docking studies highlighted the presence of another pocket, delimited by Leu69^2.46^, Ala72^2.49^ and Asp73^2.50^ of the conserved LAXADL domain of TM2, which accommodated the amide alkyl chain of the naphthalene derivative. According to this binding mode, the NH group of the indole ring of melatonin did not form any interaction with neighboring residues. The authors have taken this result as a possible explanation for the similar binding affinities seen for 2-iodomelatonin (p*K*_i_ = 10.49) and for the naphthalene derivative (p*K*_i_ = 10.53). The lower binding affinity observed for two conformationally-constrained derivatives was related to their inability to form the same pattern of polar interactions seen for melatonin.

**Figure 5:**
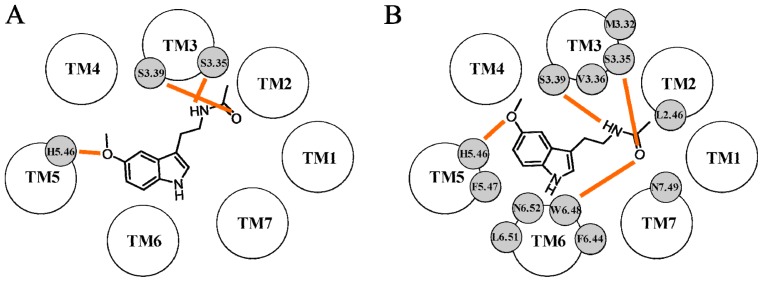
Representation of the melatonin binding scheme proposed by Chugunov *et al.* (**A**) and by Farce *et al.* (**B**) within the MT_1_ binding site. Hydrogen bond interactions are depicted with orange lines.

The first attempt to model the active state of the MT_1_ receptor was made by Chugunov *et al.* in 2006 [62]. Since bovine rhodopsin was the sole crystallized GPCR, it was taken as the template for the construction of the MT_1_ receptor (PDB ID: 1L9H [61]). Boundaries of the TM regions of the MT_1_ receptor were initially predicted with different algorithms and subsequently used as guidance during the alignment of template and MT_1_ sequences. Interestingly, in the final sequence alignment, a gap in the template and one in the MT_1_ receptor could be identified in TM4 and TM6 domains, respectively. Five MT_1_ models were initially built, and one was selected based on the analysis of spatial violations. After energy minimization, loop sequences were removed, and the TM segments were used for further studies. A visual inspection of the MT_1_ 3D structure revealed that Ser110^3.35^ and Ser114^3.39^ were oriented outside the putative binding cavity. For this reason, the authors rotated the TM3 helix in a clockwise direction (viewed from the extracellular side) to favor the exposure of the two serines. The helix was rotated from 0° (the starting conformation) to 60° with steps of 5°, yielding twelve different model structures. Melatonin was manually docked into these twelve receptors in its putative bioactive conformation, in which the amide side chain was perpendicular to the indole ring. The accommodation of melatonin into the MT_1_ binding cavity was accomplished by trying to minimize the distances between His195^5.46^ and the 5-methoxy group, as well as between the two serines on TM3 (Ser110^3.35^ and Ser114^3.39^) and the amide group. The twelve resulting complexes were then submitted to an energy minimization procedure aimed at relaxing the receptor structure around the pre-positioned melatonin molecule, and they were subsequently analyzed using different metrics. In particular, the hydrogen bond distances and the hydrogen bond angles were monitored for the three main hydrogen bond interactions, and they were compared to the ideal values of a prototypic hydrogen bond interaction. Additional quality criteria were (i) the docking score assigned to melatonin during re-docking calculations and (ii) the helix packing quality. In the best MT_1_ model, the receptor binding cavity was located in the extracellular portion of the helical bundle, lined by TM3, TM5, TM6 and TM7. A closer inspection of ligand-receptor contacts revealed that the 5-methoxy group of melatonin formed a hydrogen bond interaction with His195^5.46^, whereas the amide group was bound to the two serines located on TM3 (Figure 5A). Based on the molecular hydrophobicity potential (MHP) approach, the authors also provided a structural explanation for the MT_1_ or MT_2_ selectivity displayed by some ligands. The MHP method allows the calculation of the hydrophobic surfaces of a ligand and of its receptor through the combination of atomic hydrophobicity constants. The common hydrophobic or hydrophilic points shared by the ligand and by the surrounding receptor residues can be considered a measure of the ligand-receptor complementarity. In this case, MHP calculations performed for an MT_1_-selective benzoxazole derivative clearly showed that the hydrophobic complementarity with the MT_1_ binding site was higher than that attained with the MT_2_ subtype (the authors also built an MT_2_ receptor model, see next paragraph). The authors therefore hypothesized that the different hydrophobic complementarity observed for a ligand within the MT_1_ and MT_2_ receptor binding cavities may explain its propensity to preferentially bind to one receptor subtype.

A further attempt to model the activated state of the MT_1_ receptor was made by Farce *et al.* in 2008 [63] based on the high resolution crystal structure of rhodopsin (PDB ID: 1L9H [61]). Interestingly, two gaps could be identified in the proposed alignment between rhodopsin and the MT_1_ receptor sequences, located in TM6 and TM7. The structural quality of the minimized MT_1_ receptor was assessed through the evaluation of the hydrophobic moments of TM domains. The hydrophobic moments of the model structure closely resembled those calculated for rhodopsin, thus confirming the reliability of the orientation of the TM domains in the MT_1_ receptor. However, the residues thought to be important for ligand binding, Ser110^3.35^, Ser114^3.39^ and His195^5.46^, did not assume a favorable orientation within the putative binding site. Thus, TM3 and TM5 were rotated clockwise of 50° and 20°, respectively, to bring His195^5.46^ in the proximity of Ser110^3.35^ and Ser114^3.39^ and, consequently, to favor the formation of the predicted network of hydrogen bond interactions with the endogenous agonist. The authors hypothesized that the reorganized helical bundle could represent the activated form of the MT_1_ receptor. Melatonin was then manually docked within the MT_1_ receptor in the extracellular portion of the TM domains, in a cavity lined by TM2, TM3, TM5, TM6 and TM7. The methoxy oxygen was hydrogen bonded to His195^5.46^, and the methyl group of the 5-methoxy substituent formed hydrophobic interactions with the side chain of Phe196^5.47^ (Figure 5B). The indole ring was stabilized by a number of interactions with residues located on TM3 and TM6, including Met107^3.32^, Val111^3.36^, Leu254^6.51^ and Asn255^6.52^. While mutation of Met107^3.32^ to threonine did not alter agonist binding [31,36,64], no data are available for Val111^3.36^, Leu254^6.51^ and Asn255^6.52^. However, the corresponding amino acids in the MT_2_ receptor subtype, Val124^3.36^ and Asn268^6.52^, were found to be involved in ligand stabilization [48]. The *N*-acetyl group occupied a cavity between TM2, TM3 and TM6, where it formed an extensive network of interactions. The amide hydrogen was bound to Ser114^3.39^ and the amide oxygen to Trp251^6.48^ and Ser110^3.35^. Moreover, the methyl group of the *N*-acetyl substituent protruded into a pocket formed by Leu69^2.46^, Phe247^6.44^ and Asn291^7.49^. Gly258^6.55^ did not directly interact with the ligand, but was located at the entrance of the binding cavity, where it could influence the access of melatonergic ligands. Indeed, site-directed mutagenesis experiments showed that the substitution of Gly258^6.55^ with the bulkier threonine brought to a remarkable decrease in ligand binding [43,44]. To evaluate the ability of the MT_1_ model to discriminate between agonists and antagonists, different melatonergic ligands were docked within the putative binding region. Interestingly, while agonist molecules, including 2-iodomelatonin and agomelatine, could be easily accommodated within the binding cavity, no docking solutions were identified for the prototypic antagonist luzindole. The authors thus suggested that the failure in the docking of luzindole could be considered a positive result, which may confirm the achievement of an activated form of the MT_1_ receptor.

**Figure 6:**
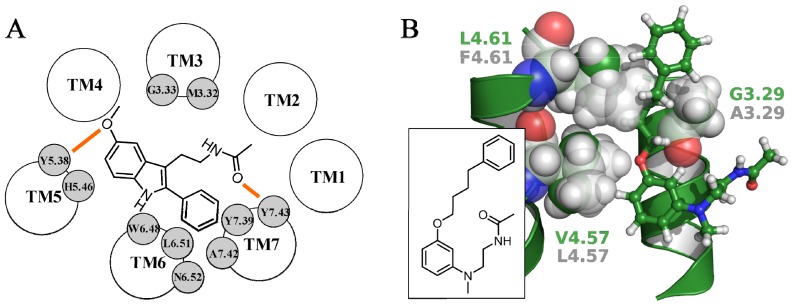
(**A**) Representation of the binding scheme of 2-phenylmelatonin within the MT_1_ binding site proposed by Rivara *et al.* Hydrogen bond interactions are depicted with orange lines; (**B**) The MT_1_-selective phenylbutyloxy derivative (ball-and-sticks representation, with green carbons) docked within the MT_1_ receptor model. Residues 3.29, 4.57 and 4.61 are represented as green (MT_1_) and transparent light gray (MT_2_) spheres. The structure of the phenylbutyloxy derivative is reported in the inset.

After the publication of the MT_1_ receptor model by Farce *et al.*, a growing number of X-ray structures of druggable GPCRs were reported in their active and/or inactive forms. The first attempt to model the MT_1_ receptor using the X-ray structure of a non-rhodopsin template was made in 2012 by our group [65]. In this study, the fully activated state of the β_2_ adrenergic receptor (PDB ID: 3P0G [66]) was used as the template structure. To adapt the binding site around a potent and bulky agonist, 2-phenylmelatonin was rigidly docked in its putative bioactive conformation, as inferred from a pharmacophore model previously developed for nonselective melatonergic agonists [67]. To favor the mutual ligand-receptor adaptation, an induced-fit docking procedure was applied, which accounted for protein flexibility during docking calculations. The resulting complexes were then evaluated on the basis of their docking score, as well as of their consistency with mutagenesis data. In particular, since a number of mutagenesis studies had shown that His^5.46^ is likely to be involved in the stabilization of the 5-methoxy group [30,31,64], the best ranked complex having the methoxy group of 2-phenylmelatonin close to His195^5.46^ was selected. The complex was then embedded in a solvated lipid bilayer to relax and to evaluate its stability during molecular dynamics (MD) simulations. 2-Phenylmelatonin was accommodated in a binding cleft located in the extracellular portion of the helical bundle, spanning from TM3 to TM7. Two main hydrogen bond interactions were identified between the methoxy oxygen and Tyr187^5.38^ and between the ligand amide oxygen and Tyr285^7.43^ (Figure 6A). Although no mutagenesis studies were performed on these two tyrosines, residue 7.43 of the MT_2_ receptor (Tyr298^7.43^) was fundamental for ligand stabilization, since its mutation to alanine brought to a complete loss of agonist binding [49]. Moreover, several mutagenesis studies performed on different class A GPCRs highlighted a key role of position 5.38 in ligand binding [68–70]. The indole ring of 2-phenylmelatonin formed extensive hydrophobic interactions with neighboring residues located on TM3 and TM5, including Gly108^3.33^ and His195^5.46^. The 2-phenyl substituent was accommodated in a hydrophobic cavity delimited by Trp251^6.48^, Leu254^6.51^, Asn255^6.52^, Tyr281^7.39^ and Ala284^7.42^, where it formed two edge-to-face interactions with Trp251^6.48^ and Tyr281^7.39^ side chains. The *N*-acetyl group pointed towards a cleft formed by the extracellular tips of TM2, TM3 and TM7, and it interacted with the side chain of Met107^3.32^. The MT_1_ receptor model was then applied to investigate the issue of ligand selectivity. The MT_1_-selective phenylbutyloxy derivative depicted in Figure 6B (p*K*_i_ (MT_1_) = 8.93; p*K*_i_ (MT_2_) = 7.04) was docked within the binding site, and the resulting complex was stabilized through MD simulations. The lipophilic phenylbutyloxy substituent undertook a number of hydrophobic interactions with residues located at the tips of TM3 and TM4 and with ECL2, including Gln101^3.26^, Gly104^3.29^, Phe105^3.30^, Val159^4.57^, Leu163^4.61^, Leu168, Gln169 and Tyr175. Interestingly, the comparison between the amino acid sequences of the extracellular ends of TM3 and TM4 in MT_1_ and MT_2_ receptors showed that the MT_1_ receptor is characterized by the presence of some smaller amino acids. Indeed, Gly104^3.29^, Val159^4.57^ and Leu163^4.61^ in the MT_1_ receptor are replaced by Ala117^3.29^, Leu172^4.57^ and Phe176^4.61^ in the MT_2_ subtype. Therefore, the presence of smaller amino acids in the MT_1_ receptor could favor the accommodation of the phenylbutyloxy substituent and, in general, of the lipophilic substituent carried by MT_1_-selective ligands.

### 3.2. MT_2_ Receptor Models

**Figure 7:**
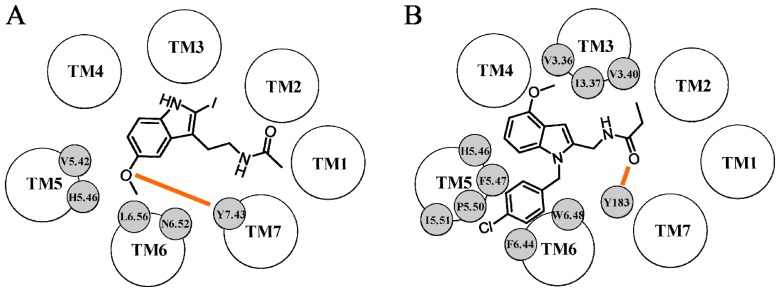
Representation of the binding scheme of 2-iodomelatonin proposed by Mazna *et al.* (**A**) and of UCM454 proposed by Rivara *et al.* (**B**) within the MT_2_ binding site. Hydrogen bond interactions are depicted with orange lines.

The first model of the MT_2_ receptor was reported in 2004 by Mazna *et al.*[49]. The rhodopsin X-ray crystal structure was used as the template (PDB ID: 1L9H [61]), and three prototypic melatonergic ligands, 2-iodomelatonin, luzindole and 4-phenyl-2-propionamidotetralin (4P-PDOT), were manually docked within the MT_2_ binding region. 2-Iodomelatonin was initially placed within the MT_2_ receptor according to the binding scheme previously proposed by Grol and Jansen for melatonin [71], which was based on the formation of a hydrogen bond between the methoxy group and His^5.46^ and of two hydrogen bonds between the amide group and Ser^3.35^ and Ser^3.39^. In the final optimized pose of 2-iodomelatonin, the 5-methoxy group was close to His208^5.46^, forming an extensive network of interactions with surrounding residues (Figure 7A). Indeed, the 5-methoxy oxygen laid within a hydrogen bond distance from the hydroxyl group of Tyr298^7.43^, which, in turn, could accept a hydrogen bond from the amide side chain of Asn268^6.52^. Interestingly, mutation of His208^5.46^ to alanine brought to a four-fold decrease in melatonin binding [32], and the substitution of Asn268^6.52^ and of Tyr298^7.43^ with alanine produced a complete loss of agonist specific binding [48,49]. The indole ring of 2-iodomelatonin formed several hydrophobic contacts with surrounding residues, such as Leu272^6.56^ and Val204^5.42^, which were shown to be important for agonist binding [49]. No hydrogen bond interactions or hydrophobic contacts were described for the *N*-acetyl group. Docking of luzindole and 4P-PDOT resulted in the absence of any polar ligand-receptor interaction within the binding cavity. Therefore, the authors hypothesized that the stabilization of these two melatonergic ligands within the MT_2_ binding cavity could be achieved through unspecific hydrophobic interactions. In the MT_2_ receptor-luzindole complex, the antagonist molecule assumed an orientation similar to that of 2-iodomelatonin, in which the indole core interacted with Leu272^6.56^ and Ala275^6.59^ side chains, and the benzyl group formed hydrophobic contacts with Leu181.

The first MT_2_ receptor model applied to address the issue of ligand selectivity was developed in 2005 by Rivara *et al.*[72]. The 3D coordinates of TM regions, ICL1 and ECL1 were modeled based on those of rhodopsin (PDB ID: 1U19 [73]), while the other loop regions were built applying a “knowledge-based” algorithm, which searches for amino acid fragments of the proper length among a Protein Data Bank-derived database. In the rhodopsin structure, a complex pattern of polar interactions between Glu122^3.37^, Trp126^3.41^ and His211^5.46^ causes a distortion of the TM5 geometry [74]. Indeed, X-ray structures of GPCRs having hydrophobic residues at position 3.37 (e.g., the κ-, μ- and δ-opioid receptors) show that TM5 is more shifted towards the helical bundle and is slightly rotated clockwise (seen from the extracellular side) compared to rhodopsin, causing a major exposure of residue 5.46 within the binding cavity. The MT_2_ receptor has a hydrophobic Ile125^3.37^ residue, and therefore, it is unlikely that the local unwinding of TM5 seen in rhodopsin could be also present in the MT_2_ receptor. For this reason, the TM5 domain of the MT_2_ model was built with an α-helix geometry and submitted to a MD simulation to relax the secondary structure, which assumed the peculiar kink associated with the presence of the highly conserved Pro212^5.50^. The MT_2_ receptor model with the “*de novo*”-built TM5 was subjected to a MD simulation to relax the whole receptor structure. To adapt the binding pocket around a potent MT_2_-selective antagonist, the indole derivative UCM454 (p*K*_i_ (MT_2_) = 8.06, Figure 7B) was docked within the MT_2_ receptor applying a set of constraints derived from mutagenesis studies and SARs, including the interaction with the conserved His^5.46^ and the arrangement of the aromatic substituent in an “out-of-plane” conformation. In the final complex, the benzene portion of the indole core interacted with the imidazole ring of His208^5.46^ through a T-shaped interaction, and the *p*-chlorobenzyl substituent was accommodated into a hydrophobic cavity located near Trp264^6.48^. To assess the reliability of this binding mode, six structurally different melatonergic antagonists were accommodated in the MT_2_ binding site in their putative bioactive conformation. The complexes were subjected to simulated annealing to favor the mutual adaptation of the ligand and the receptor binding site and then to MD simulations to check the stability of the resulting complexes. A common solution was found for all the ligand-receptor complexes, in which the ligand amide oxygen was hydrogen bonded to the hydroxyl group of Tyr183, belonging to the ECL2 segment, and the indole ring interacted with His208^5.46^. The “out-of-plane” substituent occupied a hydrophobic cleft located close to Trp264^6.48^ and lined by Val124^3.36^, Ile125^3.37^, Val128^3.40^, Phe209^5.47^, Pro212^5.50^, Ile213^5.51^, Phe260^6.44^ and Trp264^6.48^. Interestingly, the amide hydrogen did not bind any surrounding residue, supporting the hypothesis of its minor role in MT_2_ binding, as different series of potent MT_2_ ligands lacking the amide hydrogen were reported [75,76]. To investigate the receptor structural elements responsible for the MT_2_ selectivity observed for different antagonists, a comparison was made between the complexes formed by the MT_2_ and the MT_1_ receptors. A difference was seen in the amino acid composition of the hydrophobic pocket accommodating the “out-of-plane” group. In fact, the MT_2_ residues, Val128^3.40^ and Ile213^5.51^, are replaced by the bulkier Ile115^3.40^ and Met200^5.51^ in the MT_1_ receptor, leading to a smaller pocket and to disruption of polar and hydrophobic interactions with the ligands. The more favorable accommodation of the “out-of-plane” substituent typical of MT_2_-selective compounds in the hydrophobic pocket close to Trp264^6.48^ of the CWXP motif could represent, therefore, an explanation for the selectivity and for the antagonist behavior seen for these compounds.

**Figure 8:**
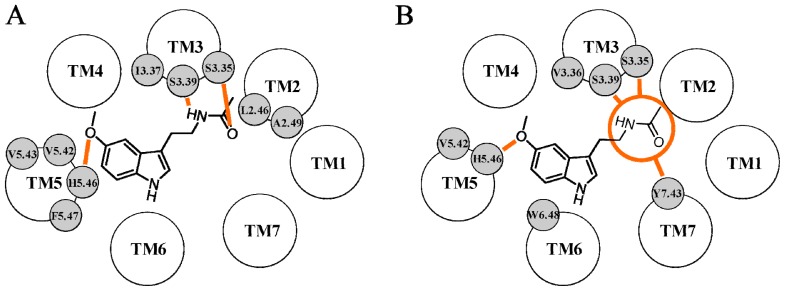
Representation of the binding scheme of melatonin proposed by Voronkov *et al.* (**A**) and Chugunov *et al.* (**B**) within the MT_2_ binding site. Hydrogen bond interactions are depicted with orange lines. The orange circle represents potential hydrogen bond interactions between the amide group of melatonin and residues 3.35, 3.39 and 7.43.

One year after the publication of an MT_1_ receptor model by the same research group, Voronkov *et al.* reported an MT_2_ receptor model based on the same rhodopsin template [77]. Interestingly, a comparison of the two sequence alignments showed a major difference concerning ECL2. Indeed, while in the case of the MT_1_ receptor, the conserved cysteine forming the disulfide bridge was correctly aligned with that of rhodopsin, in the MT_2_ receptor, the corresponding cysteine was aligned to a cysteine in the rhodopsin sequence, which is not involved in the disulfide bond. Several agonists were selected for docking simulations, and their accommodation within the binding site was inferred from site-directed mutagenesis experiments. In the final complex, the methoxy oxygen of melatonin interacted with His208^5.46^ and the amide oxygen and hydrogen atoms formed two hydrogen bonds with Ser123^3.35^ and Ser127^3.39^, respectively (Figure 8A). The methyl group of the methoxy substituent was accommodated in a hydrophobic cavity lined by Ile125^3.37^, Val204^5.42^ and Val205^5.43^ and a π–π interaction could be seen between the indole ring and the Phe209^5.47^ side chain. The methyl group of the *N*-acetyl fragment occupied a cleft lined by Leu82^2.46^ and Ala85^2.49^, located towards the intracellular end of TM2. Docking of melatonin derivatives carrying a bulky substituent at position 2, such as an iodine atom or a phenyl ring, showed that these groups could be stabilized by interactions with Trp251^6.48^ and neighboring residues, including Asp86^2.50^, Ala297^7.42^, Asn300^7.45^, Ser301^7.46^ and Asn304^7.49^. The effect of the elongation of the amide side chain in melatonin-like derivatives was also investigated. Indeed, lengthening of the acetyl group to a propionyl or to a butyryl one did not influence MT_2_ binding affinity, whereas longer acylating agents brought to a remarkable decrease in binding affinity [78]. Docking studies performed with the *n*-butyryl derivative of melatonin showed the presence of a pocket lined by Leu82^2.46^, Ala85^2.49^ and Thr168^4.53^ that could accommodate the terminal alkyl chain of the amide fragment. On the basis of these docking results, the authors proposed a structural explanation for MT_2_ selectivity. Indeed, inspection of the binding site revealed that residue 3.40 (Ile115^3.40^ in MT_1_ receptor and Val128^3.40^ in MT_2_ receptor) laid near positions 1 and 2 of the indole ring of melatonin. Thus, the smaller side chain of amino acid 3.40 in the MT_2_ receptor subtype could favor the accommodation of MT_2_-selective ligands, which often carry bulky lipophilic substituents bound to positions 1 or 2 of the indole ring. The authors also tried to explain the 60-fold decrease in binding affinity observed with the introduction of a *p*-methoxyphenyl substituent at position 1 of melatonin [79]. Docking results for this *N*-substituted derivative showed that the accommodation of the *p*-methoxyphenyl group was sterically impeded by Trp264^6.48^, Ala265^6.49^ and Leu267^6.51^ side chains. The MT_2_ receptor model was also applied to rationalize the SARs available for a series of 3-methoxyphenylalkylamides [80], in which the length of the alkyl linker varied from one to three methylene groups. Compounds with three methylenes bound melatonin receptors in the nanomolar range, while shortening of the alkyl linker to two methylene groups brought to a ten-fold decrease in binding affinity, and a further deletion of a methylene group led to micromolar affinities. Based on docking results, the authors proposed that the reduction in binding affinity observed for compounds carrying a shorter alkyl linker could be ascribed to their inability to form specific hydrogen bond interactions with key binding site residues. In fact, one and two methylene derivatives could not simultaneously bind to Ser^3.35^ and Ser^3.39^.

As previously cited, in 2006 Chugunov *et al.* explored the molecular basis of agonist recognition at both melatonin receptors [62]. The workflow applied for the building of the MT_2_ model was the same applied for the MT_1_ model, which involved an initial clockwise rotation of TM3 to favor the exposure of Ser123^3.35^, Val124^3.36^ and Ser127^3.39^ towards the putative binding cavity. It should be noted that while mutation of Val124^3.36^ altered 6-chloromelatonin binding to the MT_2_ receptor [48], mutation of Ser123^3.35^ and Ser127^3.39^ did not affect agonist binding to this receptor subtype [32]. Melatonin was manually docked into a set of MT_2_ receptor conformations characterized by different degrees of rotation of the TM3 domain, trying to minimize the distances between the 5-methoxy substituent and His208^5.46^ and between the amide group and Ser123^3.35^ and Ser127^3.39^. The MT_2_ receptor-melatonin complex, which showed (i) the lowest deviation of ligand-receptor hydrogen bond geometries, (ii) the highest score for melatonin re-docking and (iii) the best agreement between the packing of amino acids in the MT_2_ structure and that observed in a database of high-resolution X-ray structures was selected. In this complex, the methoxy oxygen was hydrogen bonded to His208^5.46^ and the methyl group interacted with Val204^5.42^, which is fundamental for ligand binding to the MT_2_ receptor [49] (Figure 8B). The indole ring of melatonin formed hydrophobic interactions with the Val124^3.36^ and Trp264^6.48^ side chains, and the amide group was stabilized by extensive hydrogen bond interactions with Tyr298^7.43^ and one of the serines, 3.35 and 3.39. In this receptor model, some residues known to be important for ligand binding laid outside the binding cavity and were not able to interact with melatonin. For example, Asn268^6.52^ and Leu272^6.56^, which were found to be crucial for agonist binding [48,49], are rotated towards the TM5-TM6 interface. Similarly to what reported for the MT_1_ receptor, the authors exploited the complementarity between the ligand and the receptor hydrophobic features to explain the MT_2_ selectivity exhibited by some melatonergic compounds. Indeed, application of the molecular hydrophobicity potential (MHP) approach on the MT_2_-selective ligand 2-benzylmelatonin highlighted that its hydrophobic complementarity was much higher with the MT_2_ receptor binding site than with the MT_1_ subtype.

**Figure 9:**
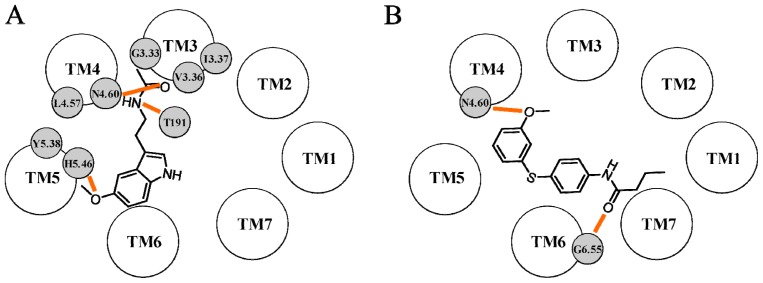
Schematic representation of the binding modes of melatonin (**A**) and of an anilide derivative (**B**) proposed by Farce *et al.* within the MT_2_ binding site. Hydrogen bond interactions are depicted with orange lines.

Farce *et al.* investigated the activation mechanism of MT_1_ and MT_2_ receptor subtypes [63]. As previously described for their MT_1_ model, the authors inserted gaps in the alignment of rhodopsin and melatonin receptor TM sequences to improve the alignment quality. The hydrophobic moments calculated for the TM portions of the MT_2_ receptor model were in good agreement with those of rhodopsin, confirming the reliability of the relative orientation of TM helices. Information from mutagenesis experiments was used to guide the docking of melatonin, and Asn175^4.60^ and His208^5.46^ were chosen as the main hydrogen bonding partners. TM5 helix of the MT_2_ receptor model was rotated clockwise to favor the exposure of His208^5.46^ towards the binding region and, consequently, towards two residues important for ligand binding, Val258^6.42^ and Ala275^6.59^. Moreover, it was found that a counterclockwise rotation of TM4 of 26° favored the accommodation of melatonin within the binding cavity without severe steric clashes with surrounding amino acids. The authors thus proposed that the final model conformation achieved by the rotation of TM domains could correspond to the putative activated form of the receptor. In the final MT_2_ receptor-melatonin complex, the methoxy oxygen of melatonin was hydrogen bonded to the imidazole ring of His208^5.46^, and the methyl group formed hydrophobic interactions with Leu172^4.57^ and Asn175^4.60^ (Figure 9A). Tyr200^5.38^ laid close to the 5-methoxy group of melatonin, with its hydroxyl group placed within a hydrogen bond distance from the methoxy oxygen of melatonin. The amide group was located towards the extracellular side of the receptor. The amide oxygen and hydrogen atoms interacted with Asn175^4.60^ side chain and with the backbone carbonyl group of Thr191 (ECL2), respectively, whereas the terminal methyl group was in contact with the side chain of Leu181 (not shown in Figure 9A). The indole ring of melatonin was accommodated in a pocket lined by TM3, TM4, TM5 and TM6, and it was stabilized by hydrophobic contacts with neighboring residues, such as Gly121^3.33^, Val124^3.36^ and Ile125^3.37^. Different residues important for melatonin binding defined the binding pocket, but they did not directly interact with the agonist molecule, including Asn268^6.52^ and Tyr298^7.43^. Docking studies were performed to evaluate the ability of the model to discriminate between agonist and antagonist molecules. The antagonist luzindole could not be accommodated within the MT_2_ binding site, and as in the case of the MT_1_ model, the authors suggested that the inability of the MT_2_ structure to accommodate an antagonist molecule could be an indication of its active state. Conversely, the agonists, 2-iodomelatonin and agomelatine, could be easily docked within the MT_2_ binding site, where they formed the same network of hydrogen bond interactions seen for the endogenous agonist. However, MT_2_ agonists characterized by different chemical scaffolds had completely different orientations within the binding site and, consequently, a diverse pattern of interactions with binding site residues. As an example, the methoxy and the amide oxygens of the anilide derivative depicted in Figure 9B were hydrogen bonded to Asn175^4.60^ and Gly271^6.55^, respectively, which did not interact with melatonin. Since all the docked MT_2_ agonists share the pharmacophoric features of melatonergic ligands, they are probably characterized by the same binding mode. The authors proposed that the remarkable differences observed between the binding modes of chemically different MT_2_ receptor agonists may be due to the rigid receptor model used for docking studies. Indeed, the accommodation of different chemical scaffolds may require slight structural rearrangements of the binding site cavity, which could not be achieved with a “frozen” receptor model.

The importance of conserved proline residues for the functional state of the MT_2_ receptor was investigated by Mazna *et al.*[81]. In this study, site-directed mutagenesis was applied to several proline residues located within the TM domains, which were replaced by either alanine, glycine or valine. The NAXXY motif, which is fully conserved among the melatonin receptor subfamily, was replaced by the NPXXY sequence found in the majority of class A GPCRs. The 3D structure of the wild-type MT_2_ receptor was built starting from the X-ray structure of bovine rhodopsin (PDB ID: 1U19 [73]). The effects of proline substitution were investigated by means of MD simulations of the wild-type receptor and of the MT_2_ mutants embedded in a solvated lipid bilayer. Pro174^4.59^ is fully conserved within the melatonin receptor subfamily, and its mutation to alanine brought to a complete loss of 2-iodomelatonin binding. This residue was located at the extracellular tip of TM4, where it delimited the upper region of the binding cavity. Although no remarkable structural changes could be identified during MD simulation of the Ala174^4.59^ mutant compared to the wild-type receptor, the authors proposed that the substitution of this residue may influence the network of interactions at the upper portion of the binding crevice, leading to the loss of agonist binding. In contrast with what observed for Pro174^4.59^, replacement of Pro212^5.50^ with alanine remarkably reduced receptor signaling, but did not alter 2-iodomelatonin binding affinity. MD simulation of the Ala212^5.50^ mutant showed a distorted geometry of the TM5 domain one turn after position 5.50, which was not observed in the simulation of the wild-type receptor. Consequently, the authors hypothesized that the bulge observed in the TM5 domain of the Ala212^5.50^ mutant may influence the conformation of binding site residues involved in the signal transduction mechanism, including His208^5.46^ and Trp264^6.48^. Pro266^6.50^ is another proline highly conserved within the melatonin receptor subfamily, as well as among class A GPCRs, and substitution with the smaller alanine brought to a complete loss of 2-iodomelatonin binding. Inspection of MD trajectories obtained for the Ala266^6.50^ mutant highlighted a disruption of the helix geometry of the extracellular portion of TM6. Since this stretch delimited the binding cavity and was tightly packed with neighboring helices, the authors suggested that the effect on agonist binding seen for the Ala266^6.50^ mutant may be due to an unfavorable packing of binding site residues. 2-Iodomelatonin was not able to bind to the Pro305^7.50^ mutant in the conserved NAXXY motif, although the receptor was found to be correctly expressed in the cell membrane. MD simulation of the Pro305^7.50^ mutant showed a disruption of the helical structure of the TM7 domain, which alters the pattern of interactions occurring between TM1, TM2, TM6 and TM7. Thus, the receptor structure bearing the mutation Pro305^7.50^ was characterized by a remarkable rearrangement of the binding site region compared to the wild-type, which may explain the loss of agonist binding observed for this specific mutant. When docked in the wild-type receptor, melatonin was surrounded by Val204^5.42^, His208^5.46^, Asn268^6.52^ and Trp264^6.48^, but no description of the interactions with the binding site residues is reported in the article.

**Figure 10:**
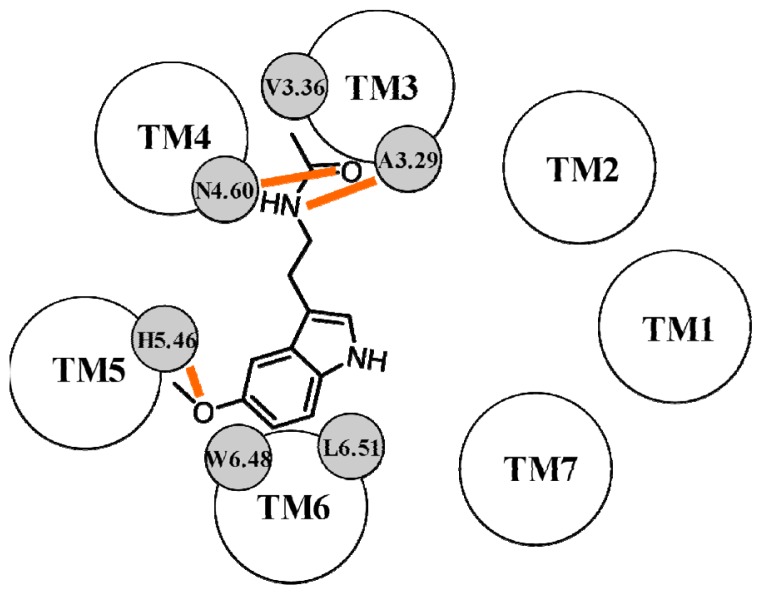
Representation of the binding scheme of melatonin proposed by Zefirova *et al.* within the MT_2_ binding site. Hydrogen bond interactions are depicted with orange lines.

Zefirova *et al.* first exploited the fully activated form of rhodopsin (PDB ID: 3DQB [82]) to build the agonist-bound form of the MT_2_ receptor [83]. Only the TM portions were included in the final MT_2_ receptor structure, as loop regions were excluded from the homology modeling procedure. When docked within the binding site cavity, melatonin interacted with His208^5.46^ and Asn175^4.60^ through its 5-methoxy and amide oxygens, respectively (Figure 10). The indole ring formed hydrophobic interactions with surrounding residues, including Val124^3.36^, Trp264^6.48^ and Leu267^6.51^, and the amide hydrogen was bound to the backbone carbonyl group of Ala117^3.29^. The authors tried to exploit the putative activated form of the MT_2_ receptor to rationalize the different binding affinities seen for three conformationally constrained derivatives. Docking studies showed that the least potent compound had unfavorable steric clashes with Tyr294^7.39^. The authors, therefore, hypothesized that this close contact might bring unfavorable structural changes of the binding crevice and, consequently, a decrease in binding affinity.

## 4. Conclusions and Perspectives

Since the publication of the first GPCR X-ray structure in 2000, a number of MT_1_ and MT_2_ receptor models have been proposed, mostly trying to hypothesize the interactions of melatonin with amino acid residues of the putative binding site. A comparison between MT_1_ and MT_2_ 3D structures shows that the proposed accommodation for melatonin seems more conserved among MT_1_ models. This is probably related to the information for each receptor subtype coming from mutagenesis studies. Indeed, while only three residues, Ser110^3.35^, Ser114^3.39^ and His195^5.46^, have been shown to be important for ligand binding at the MT_1_ receptor, mutagenesis experiments at the MT_2_ receptor identified a substantial number of important residues located in almost all TM domains and in loop regions. Consequently, while melatonin was often bound to the three aforementioned residues in MT_1_ receptor models, alternative orientations of the endogenous ligand led to different interaction patterns at the MT_2_ receptor, providing a wide array of putative binding modes. Although information provided by mutagenesis studies do not allow the identification of the amino acid counterparts for melatonin, the definition of an unambiguous pattern of ligand-receptor interactions at both MT_1_ and MT_2_ subtypes is also hampered by other elements. Indeed, the reliability of melatonin receptor models is limited by the low degree of sequence identity shared with available GPCR template structures. The significant differences in the amino acid sequence between melatonin receptors and template structures probably reflect notable dissimilarities in secondary and tertiary structures, including shifts and bulges of the TM domains, as well as in the architecture and organization of loop portions. For example, in a number of MT_1_ and MT_2_ receptor models built starting from the X-ray structure of bovine rhodopsin, the ECL2 portion sterically hampered the accommodation of ligands within the binding site. This is due to the peculiar geometry of the ECL2 of rhodopsin, which seals the retinal binding site and prevents the penetration of solvent molecules within the lipophilic binding pocket. As a consequence, in many melatonin receptor models, ECL2 was either deleted or rearranged without a template structure to guide the structural modification [58,62,83]. Probably due to the aforementioned difficulties, structure-based drug design has never been a viable option, as this approach strongly depends on the availability of a reliable receptor structure and of a well-characterized pattern of ligand-receptor interactions. Thus, melatonin receptors have been mainly applied in retrospective analyses to rationalize mutagenesis data and to reproduce SARs.

Despite a decade of progress in the field of GPCR structural biology, the modeling of melatonin receptors can still be considered a challenging task. This is, in general, true for “remote-template” GPCRs (*i.e.*, those receptors sharing a low sequence identity with available template structures), whose 3D structures have been hardly predicted by homology modeling procedures [16–18]. However, although the modeling of these receptors has to face the uncertainties related to available structural templates, the growing body of experimental and ligand-based information could be used to compensate the phylogenetic distance between the template and the receptor model and, consequently, may improve the receptor model quality. Accordingly, several modeling studies of rhodopsin-like GPCRs have highlighted the importance of incorporating a knowledge-guided refinement of the receptor structure to obtain reliable receptor-ligand complexes [84–86]. This approach was also applied to melatonin receptors. Indeed, incorporation of information obtained from pharmacophore models and SARs led to MT_1_ and MT_2_ receptor models, which could provide a structural explanation for ligand selectivity and intrinsic activity [65,72]. Modeling of “remote-template” receptors should also benefit from the application of advanced computational techniques. Nowadays, long-timescale MD simulations have become viable for receptor-membrane systems, allowing for an extensive structural refinement of the receptor model and providing the possibility to describe the conformational changes occurring upon ligand binding [87]. Recently, remarkable advances have also been reported in the field of GPCR loop prediction. Loop regions usually show a low degree of sequence conservation between different GPCR families, making loop modeling a complicated task. Modeling of such sequences is even more difficult, due to the extensive pattern of interactions that they can form with the ligand and with the neighboring loop segments. For these reasons, a lot of efforts have been made to improve the quality of loop predictions, leading to novel algorithms and approaches able to correctly reproduce the architecture of GPCR loop portions [88,89].

In conclusion, modeling of “remote-template” receptors could take advantage of different resources, such as mutagenesis data, ligand-based information, computational techniques and of the exponential growth in the number of available GPCR X-ray crystal structures. The boost in the comprehension of the GPCR “machinery” at a molecular level and the exploitation of available information could lead to a new generation of reliable GPCR homology models, which, hopefully, could be actively used in structure-based drug design campaigns.
